# Overexpression of Rice Glutaredoxin *OsGrx_C7* and *OsGrx_C2.1* Reduces Intracellular Arsenic Accumulation and Increases Tolerance in *Arabidopsis thaliana*

**DOI:** 10.3389/fpls.2016.00740

**Published:** 2016-06-01

**Authors:** Pankaj K. Verma, Shikha Verma, Veena Pande, Shekhar Mallick, Rudra Deo Tripathi, Om P. Dhankher, Debasis Chakrabarty

**Affiliations:** ^1^Genetics and Molecular Biology Division, Council of Scientific and Industrial Research-National Botanical Research InstituteLucknow, India; ^2^Department of Biotechnology, Kumaun UniversityNainital, India; ^3^Environmental Biotechnology Division, Council of Scientific and Industrial Research-National Botanical Research InstituteLucknow, India; ^4^Stockbridge School of Agriculture, University of MassachusettsAmherst, Massachusetts

**Keywords:** arsenic, GSH, *OsGrxs*, glutaredoxin, *Oryza sativa*, aquaporin

## Abstract

Glutaredoxins (*Grxs*) are a family of small multifunctional proteins involved in various cellular functions, including redox regulation and protection under oxidative stress. Despite the high number of *Grx* genes in plant genomes (48 *Grxs* in rice), the biological functions and physiological roles of most of them remain unknown. Here, the functional characterization of the two arsenic-responsive rice Grx family proteins, OsGrx_C7 and OsGrx_C2.1 are reported. Over-expression of *OsGrx_C7* and *OsGrx_C2.1* in transgenic *Arabidopsis thaliana* conferred arsenic (As) tolerance as reflected by germination, root growth assay, and whole plant growth. Also, the transgenic expression of *OsGrxs* displayed significantly reduced As accumulation in *A. thaliana* seeds and shoot tissues compared to WT plants during both AsIII and AsV stress. Thus, *OsGrx_C7* and *OsGrx_C2.1* seem to be an important determinant of As-stress response in plants. *OsGrx_C7* and *OsGrx_C2.1* transgenic showed to maintain intracellular GSH pool and involved in lowering AsIII accumulation either by extrusion or reducing uptake by altering the transcript of *A. thaliana AtNIPs*. Overall, *OsGrx_C7* and *OsGrx_C2.1* may represent a Grx family protein involved in As stress response and may allow a better understanding of the As induced stress pathways and the design of strategies for the improvement of stress tolerance as well as decreased As content in crops.

## Introduction

Glutaredoxins (Grxs) are ubiquitous low molecular weight, cysteine-rich proteins that take part in diverse cellular processes including maintenance and regulation of cellular redox state, iron homeostasis and redox-dependent signaling pathways (Holmgren, 1989; Holmgren and Aslund, 1995; Lillig et al., 2008). Various *Grx* genes had been identified in both eukaryote and prokaryote. Based on the predicted amino acid sequences and arrangement of cysteine residues in active-site motifs, all *Grx*s are divided into three major classes CPYC-, CGFS-, and CC-type. The CC-type class has been identified in land plants only whereas others are found in all organisms from prokaryotes to eukaryotes (Rouhier et al., 2004, 2006; Garg et al., 2010).

Glutaredoxins are GSH-dependent redox enzymes that catalyze the reduction of disulfides through reduced GSH in a coupled system with NADPH and GR (Fernandes and Holmgren, 2004). Besides the traditional roles of thiol-disulfide oxidoreductases in oxidative stress responses, many other functions of *Grxs* were also reported, including roles in iron homeostasis, iron-sulfur cluster biosynthesis, and stress-related redox sensor (Michelet et al., 2008; Dalle-Donne et al., 2009; Rouhier et al., 2010; Zaffagnini et al., 2012). Protein-*S*-glutathionylation is also a reversible post-translational modification of thiol groups with GSH, which provides protection to the cysteine residues of proteins from irreversible oxidation and may also alter the activity of many proteins either positively or negatively (Gallogly and Mieyal, 2007). Protein glutathionylation in plants is favored by the condition of enhanced reactive oxygen species (ROS) production, where glutathionylation is a mechanism for redox regulation and signaling. Grxs are efficient catalysts for the de-glutathionylation reaction, although the fundamental mechanism of glutathionylation *in vivo* remains obscure (Dalle-Donne et al., 2009; Gao et al., 2010). Due to the involvement of Grxs in many cellular processes, these enzymes have been implicated in a variety of significant physiological and pathological processes (Lillig et al., 2008). In contrast, to the plenty of reports about *Grx*s gene structure and biochemical activity, the functions of plant *Grx*s are still elusive. It was known that abiotic stresses including metals and metalloids (As) exposure increase ROS production. The different ROS, including superoxide ($O_{2}^{•–}$), hydrogen peroxide (H_2_O_2_), singlet oxygen (O_2_), and hydroxyl radicals (OH^∙^), may lead to unspecific oxidation of proteins and membrane lipids and also causes DNA injury (Pastori and Foyer, 2002; Apel and Hirt, 2004). To cope with As stress-induced oxidative stress plants have evolved ROS-scavenging enzymes, such as superoxide dismutase (SOD), catalase (CAT), ascorbate peroxidase (APX) and complex antioxidant systems, such as glutaredoxin and thioredoxin (Apel and Hirt, 2004).

High As concentrations in groundwater is a serious problem in several countries, including Bangladesh, China, India, Japan, and some parts of the United States. Crops including rice grown on soils irrigated with As-contaminated water accumulate high amount of As in their edible parts. Thus, the As contaminated rice becomes a primary source for As contamination in human and causes a serious health risk and many diseases including cancer, skin lesions (Anawar et al., 2002; Meharg and Rahman, 2003; Das et al., 2004). Also, the rice stem and husk used as fodder for animals can make a route of As entry in human. So, there is an urgent need to identify and develop an efficient strategy to minimize As accumulation in plant parts (seeds and shoot). Several studies conducted on As tolerance and metabolism in plants, such as screening of low As accumulating rice varieties, studies on transporters and genes involved in As uptake and partitioning in rice plants. Earlier reports on As uptake and translocation in plants confirmed that various genes were involved in uptake of AsV and AsIII, reduction of AsV to AsIII, transportation, chelation, vacuolar sequestration and extrusion of AsIII. Arsenate enters into the cell via OsPHT1;1 (Kamiya et al., 2013), OsPHT1;8 (Wu et al., 2011) in rice and AtPHT1;1, AtPHT1;4, AtPHT1;5, AtPHT1;7, AtPHT1;8, AtPHT1;9 in *A. thaliana* (Shin et al., 2004; Catarecha et al., 2007; Remy et al., 2012; LeBlanc et al., 2013; Fontenot et al., 2015). Whereas, the AsIII can enter and transported via aquaporins such as AtNIP1;1, AtNIP1;2, AtNIP5;1 (Kamiya and Fujiwara, 2009), AtNIP3;1 (Xu et al., 2008), AtNIP6;1 (Bienert et al., 2008), AtNIP7;1 (Isayenkov and Maathuis, 2008) in *A. thaliana* and OsNIP1;1, OsNIP2;2 (OsLsi6), OsNIP3;1 (Ma et al., 2008), OsNIP3;2 (Bienert et al., 2008), OsNIP3;3 (Katsuhara et al., 2014) in rice. OsLsi1 facilitates the influx of AsIII and organic arsenical (DMA, MMA) whereas, OsLsi2 involved in eﬄux and xylem loading (Ma et al., 2008). In earlier studies, As tolerance mechanism in plants mainly focuses on the uptake of As (AsIII and AsV) and formation of AsIII complex with phytochelatins, metallothionein, and glutathione to transport in cell vacuoles (Tripathi et al., 2007). However, only a few studies conducted on AsIII tolerance mediated by plant *Grxs* were reported. *Pteris vittata Grx* (*PvGrx* 5-6) when overexpressed in *A. thaliana* increases As tolerance and decreases As accumulation in transgenic plants by regulating intracellular AsIII levels during AsV stress. However, the exact mechanism was not identified (Sundaram et al., 2008, 2009; Sundaram and Rathinasabapathi, 2010). In an earlier study, we also reported that two glutaredoxins were up-regulated during both AsV and AsIII stress and might have a role in As tolerance (Chakrabarty et al., 2009; Rai et al., 2011; Dave et al., 2013; Dubey et al., 2015). Also, the upregulation of *OsGrxs* was found in both As sensitive and tolerant varieties, but the expression of *OsGrxs* was higher in sensitive varieties along with various other genes (Dave et al., 2013). Recently, we showed that the rice glutaredoxin (*OsGrx*) genes play a role in detoxification of arsenicals by GSH recycling and regulating intracellular AsIII levels by indirectly or directly modulating the aquaglyceroporins (AsIII channels) that conduct AsIII entry or eﬄux from yeast cells (Verma et al., 2016).

Here, we reported the increased As stress tolerance in *OsGrxs* expressing transgenic *A. thaliana* lines compared to wild-type (WT) lines, thus providing the evidence for the involvement of rice glutaredoxin (*OsGrxs*) genes in As tolerance in plants. Furthermore, during As stress, expression of the certain *A. thaliana* aquaporin genes (NIPs) was up-regulated in *OsGrxs* expressing transgenic plants compared with WT controls. Thus, these findings suggest a specific protective role of a redox protein against As stress and provide a genetic engineering strategy to improve safer crop production.

## Materials and Methods

### Plant Material

Rice (*Oryza sativa*, accessions IC-115730, and IC-3470072) seeds were germinated and let to grow in hydroponic conditions. After 1-week, seedlings were exposed to Na_2_HAsO_4_ (10 and 25 μM) and NaAsO_2_ (10 and 25 μM) in the hydroponic medium for another week (Dave et al., 2013).

*Arabidopsis thaliana* (ecotype Columbia) were grown in plastic containers, using soilrite and irrigated by Hogland’s nutrient solution. The plants were grown under 16 h light photoperiod of 150 mmol s^-1^ m^-2^ supplied by cool white fluorescent lights at 23°C constant temperature. Upon reaching 5–10 cm length, the primary inflorescences were clipped once to favor the growth of multiple secondary bolts for plants to be used for *Agrobacterium*-mediated transformation (Clough and Bent, 1998).

### Expression Analysis of Two As Responsive *OsGrxs* During As Stress

The expression of *OsGrxs* in Na_2_HAsO_4_ (10 and 25 μM) and NaAsO_2_ (10 and 25 μM) stress were examined. RNA was extracted from rice roots using Qiagen RNeasy (Qiagen, USA) and cDNA was prepared using RevertAid First Strand cDNA synthesis kit (Thermo Scientific, USA). This cDNA was used as a template for real-time PCR described in the latter sections. Primers used in this study were listed in Supplementary Table S1.

### Construction of Expression Vector and *Agrobacterium*-Mediated Transformation

The open reading frame of the *OsGrx_C7* (312 bp) and *OsGrx_C2.1* (402 bp) cDNA clone was amplified using the gene specific primers. The PCR product cloned in the pTZ vector to obtain pTZ-*OsGrx_C7* and pTZ-*OsGrx_C2.1* and verified by sequencing. Both *OsGrxs* were cloned in *Xba*I – *Sac*I linearized plant expression vector pBI121 to derive pBI121-*OsGrx_C7* and pBI121-*OsGrx_C2.1* (Supplementary Figure S1A). The pBI121-*OsGrx_C7*, pBI121-*OsGrx_C2.1* and pBI121 were transferred into *Agrobacterium tumefaciens* GV3101 strain via freeze-thaw method (Jyothishwaran et al., 2007). Plants were inoculated with *Agrobacterium tumefaciens* strain GV3101, carrying pBI121-*OsGrx_C7*, pBI121-*OsGrx_C2.1* or pBI121 by the floral dip method (Clough and Bent, 1998). The aerial floral parts of the plants were dipped for 1 min with gentle shaking in 100 mL of 5% (w/v) sucrose with *Agrobacterium tumefaciens* cells at 0.8 A_600_ and 0.02% (v/v) of the surfactant Silwet L-77 (Union Carbide, Danbury, CT, USA). After inoculation, plants were covered with a transparent plastic to maintain humidity for 24 h and then kept at growth chamber till seed set. Putative transformants (T1) were selected based on their resistance to kanamycin (50 mg L^-1^) in the ½ × MS medium with 2% sucrose and 0.8% (w/v) agar. Further, the presence of the transgene was verified using a PCR procedure on isolated genomic DNA as a template with *OsGrxs* gene-specific primers. The putative transgenic plants were grown in controlled environments in growth chamber and seeds were collected from individual plants. From the T2 seeds, several plants were selfed and their progeny analyzed for homozygosity. Homozygous T3 lines identified from these analyzes were used for evaluations of transgene expression and functional validation.

### Quantitative Reverse Transcriptase-PCR (qRT-PCR)

Transcript quantification was done by qRT-PCR using 7500 fast real-time PCR system (Applied Biosystems, USA). Total RNA, isolated using Qiagen RNeasy (Qiagen, USA) was used for cDNA synthesis. cDNA synthesis was done by using RevertAid First Strand cDNA Synthesis Kit (Thermo Scientific, USA). This cDNA was used as a template for quantifying the transcript level of *OsGrx*s using the gene-specific primers using 2 × Master SYBR^®^ Green (Applied Biosystem, USA). Primers for an *AtTub6* were included as an internal control. The relative shift in the threshold cycle (ddCt) value was determined by subtracting the average dCt of *Grx* – average dCt of tubulin (*n* = 3). Moreover, expression was calculated by the 2ˆ-ddct method.

### Glutaredoxin Assay

Soluble protein was extracted from leaves using method previously described by Fouad and Rathinasabapathi (2006). Grx activity was determined with a coupled enzyme reaction described by Holmgren (1989). In this assay, NADPH-dependent reduction of 2-hydroxyethyl disulfide (HED) in the presence of glutathione reductase (GR) was monitored at 340 nm using a UV-visible spectrophotometer (Perkin Elmer, USA). The assay mixture contained 100 mg mL^-1^ of bovine serum albumin, 1 mM GSH, 6 μg mL^-1^ yeast GR, 0.4 mM NADPH, 0.1 M Tris–HCl, 2 mM ethylene diamine tetraacetic acid (EDTA), pH 8.0, and 0.7 mM HED in a total volume of 800 μL. Non-enzymatic NADPH-dependent reduction of HED was measured for the background. Total protein was estimated by Bradford method. The enzyme activity was expressed as nmol mg^-1^ protein min^-1^.

### Evaluation of As Tolerance of *OsGrxs* Transformed *A. thaliana*

Arsenic resistance during germination was evaluated by plating the transgenic seeds in ½ × MS medium containing 2% (w/v) sucrose and 0.8% (w/v) agar with or without 25 μm sodium arsenite and 250 μm sodium arsenate. The seeds were surface sterilized using 2% (v/v) sodium hypochlorite and washing thoroughly with sterilized water. Approximately 100 seeds were plated and then kept at 4°C for 48 h for stratification, and then transferred at 23°C for 3 days under light for calculating the germination rate under control and the As treatment conditions.

Root growth was evaluated by germinated seed on ½ × MS agar medium. Three-day old seedlings were transferred onto vertical plates containing ½ × MS agar medium, containing varying concentrations of sodium arsenate (50, 100, 250, and 500 μM) or sodium arsenite (5, 10, 25, and 50 μM). By marking and measuring the root tip at the beginning and end of a 10 days period, root length and fresh weight were calculated.

To evaluate the whole plant As tolerance, *OsGrxs* expressing transgenic lines and WT were grown in individual containers under identical conditions. *A. thaliana* plants were irrigated with or without sodium arsenate (250 μM) and sodium arsenite (25 μM) in Hoagland nutrient solution (Hoagland and Arnon, 1950). The As-containing medium was added to the potting medium without touching the aerial portion of the plants. After 6 weeks, growth difference was observed by measuring the aboveground biomass dry weight of completely dried samples.

### Measurement of As Accumulation in Transgenic *A. thaliana*

Arsenic accumulation for short-term was assayed by growing *A. thaliana* plant for 7 days in hydroponic medium with or without 25 μM AsIII and 250 μM AsV. Samples were washed with 10 mM EDTA and distilled water, dried, mineralized in HNO_3_ and As was analyzed using ICP-MS. Simulated irrigation experiment assayed long-term exposure effect on the plant’s aerial parts. Fourteen-days old plants were transferred in soilrite and grown under controlled environments, watered with nutrients solution containing 25 μM AsIII and 250 μM AsV every week till plant completes its life cycle. Subsequently, plants were thoroughly rinsed with 10 mM EDTA and distilled water, dried, and digested in HNO_3_ and amount of As in the samples was determined using ICP-MS (Perkin Elmer, USA) (Supplementary Table S2).

### Expression Analysis of *A. thaliana* Aquaporin Genes

Seeds of *A. thaliana* WT (Columbia-0) and transgenic lines were sterilized and germinated as described in previous sections. After 14 days, plants were transferred to hydroponics culture medium with 10 μM, 25 μM AsIII and 100 μM, 250 μM AsV under controlled environments (16 h light: 8 h dark cycle, 22°C, 150 mMm^-1^s^-1^ light intensity and 70% relative humidity). After 7 days, plants were harvested, root and shoot tissues used for RNA isolation and extraction of total protein.

Total RNA was extracted from *A. thaliana* plants using RNeasy Plant Mini Kit according to the manufacturer’s instructions (Qiagen). The qRT-PCR were performed using 2 × SYBR Green Master Mix and ABI7500 fast (Applied Biosystem, USA) in a total volume of 20 μl. The reaction contains 1 μl of each primer (5 μM), 7 μl of nuclease-free water, and 10 μl of 2× Master SYBR^®^ Green (Applied Biosystem, USA) and cDNA as template. Reaction condition at 95°C for 10 min followed by 40 cycles of 95°C for 10 s, 60°C for 10 s, 72°C for 15 s. The reaction without template designated as a negative control in the same PCR run for each primer pair. For each of the two independent RNA extractions, measurements of gene expression were obtained in triplicate.

### Determination of Total GSH and GSSG in Transgenic *A. thaliana* Lines under As Stress

*Arabidopsis thaliana* plants were grown as described in above section. Plants were washed and re-suspended in 100 mM potassium phosphate buffer (pH 7.8), with 5% 5-sulfosalicylic acid, centrifuged for 30 min at 5000 × *g* at 4°C and the supernatant was used to determine total free GSH concentration.

The reaction mixture contains the cell extract (10 μL), 0.50 mM DTNB, and 0.25 mM NADPH in 100 mM potassium phosphate buffer with 5 mM EDTA, pH 7.8. The reaction was started by adding 0.2 U of GR and kinetics of DTNB conversion to TNB were followed spectrophotometrically at 412 nm. GSH concentrations were calculated from standard curves obtained with various GSH and GSSG concentrations based on the rate of TNB formation and expressed as nanomoles of GSH/mg of protein. GSH: GSSG ratios were calculated using the equation GSH/GSSG = 2(total GSH-GSSG)/GSSG (Rahman et al., 2007).

### Data Analysis

Quantitative data were analyzed by analysis of variance using Graph Pad Prism program. All values reported in this work are means of at least five individual replications unless mentioned otherwise. Experiments were repeated three times. Results were expressed as means followed by corresponding standard errors.

## Results

### Two Rice Glutaredoxin (*OsGrx*) Genes Induced during As Stress

**FIGURE 1:**
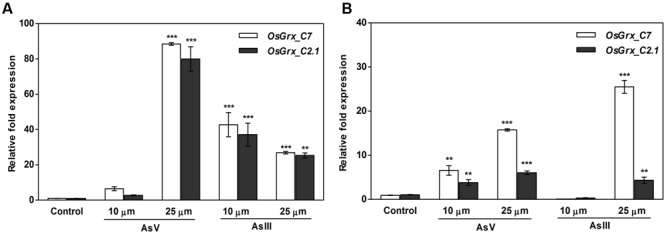
**Quantitative Reverse Transcriptase-PCR (qRT-PCR) analysis of As-responsive glutaredoxin gene revealed that both *OsGrxs* upregulated under As stress.** For qRT-PCR analysis, the rice (*Oryza sativa*) seedlings of **(A)** sensitive (IC 115730) and **(B)** tolerant (IC 340072), treated with AsV and AsIII were used for RNA isolation. Expression analysis was performed using *OsGrx_C7* and *OsGrx_C2.1* gene specific primers and rice actin as an internal control. Asterisks indicate significant differences (^∗∗∗^*P* < 0.001, ^∗∗^*P* < 0.01). Error bar mean ± SE.

Global gene expression analysis of rice during As stress showed up-regulation of two genes, LOC_Os01g27140 (*OsGrx_C7*), and LOC_Os01g40500 (*OsGrx_C2.1*) designated as members of Grx protein family in rice (Chakrabarty et al., 2009). The expression of *OsGrx*s was analyzed in As tolerant (IC-340072) and sensitive (IC-115730) rice cultivars (Dave et al., 2013) under AsIII stress. To further extend this analysis, the qRT-PCR analysis done with a different set of As treated samples and results suggested that the expression of *OsGrx_C7* and *OsGrx_C2.1* get induced after exposure to both AsIII and AsV. Both *OsGrxs* show high mRNA accumulation under AsV stress compared to AsIII. However, sensitive cultivar (IC-115730) showed higher expression level as compared to tolerant cultivar. Also, *OsGrxC_7* have higher expression level than *OsGrxC_2.1* under As stress (**Figure 1**). Thus, our result indicated that both *OsGrx*s might involve directly or indirectly in As tolerance and metabolism.

### Overexpression of *OsGrx*s in Transgenic *A. thaliana*

Transgenic *A. thaliana* lines were obtained, that ectopically express *OsGrx_C7* and *OsGrx_C2.1* under the transcriptional control of the CaMV 35S promoter. Expression of the transgene was analyzed in several independent lines. The transgenic lines showed varying expression levels of *OsGrx_C7* and *OsGrx_C2.1*. Three of each *OsGrxs* expressing lines with similar expression pattern were selected for further analysis (Supplementary Figure S1B). The growth and development of transgenic lines and WT were comparable in pots or on half-strength MS plates.

### *OsGrxs* Expressing Transgenic *A. thaliana* Lines Display Tolerance to AsIII and AsV

**FIGURE 2:**
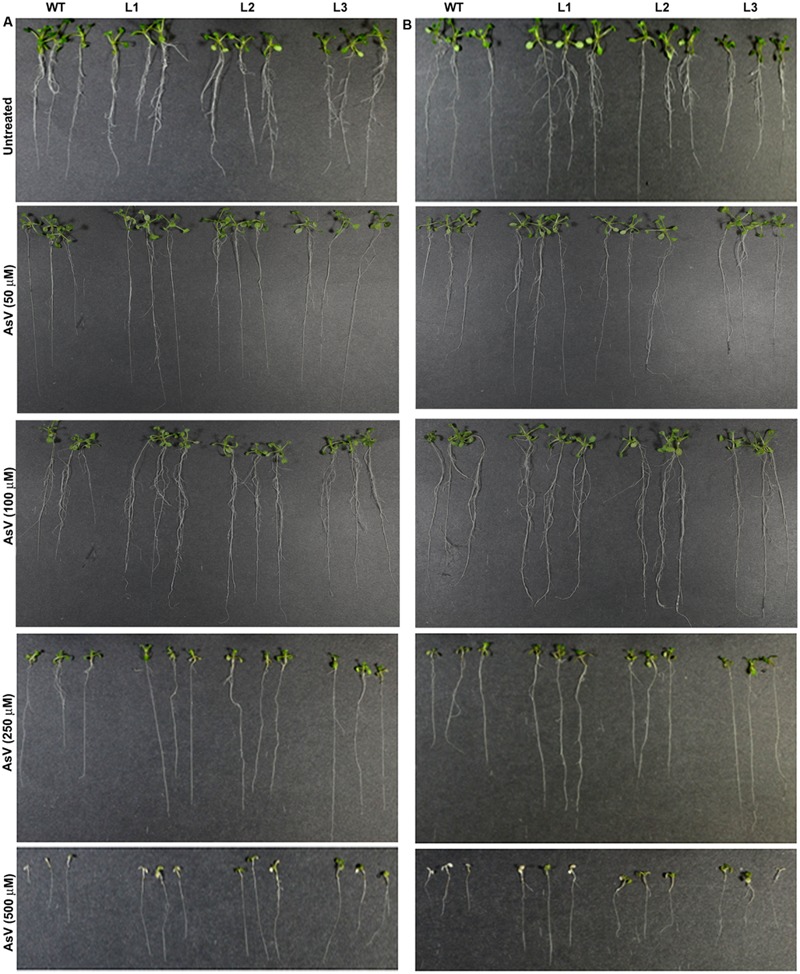
**Enhanced arsenate (AsV) tolerance in *A. thaliana* plants transformed with **(A)***OsGrx_C7* and **(B)***OsGrx_C2.1.*** Phenotypic changes in WT and transgenic *A. thaliana* plants carrying *OsGrx_C7* and *OsGrx_C2.1* observed after grown vertically on plates of ½ × MS medium without As and with 50 μM AsV, 100 μM AsV, 250 μM AsV, and 500 μM AsV, for 10 days. (*n* = 5 plants per treatment per line and repeated five times).

**FIGURE 3:**
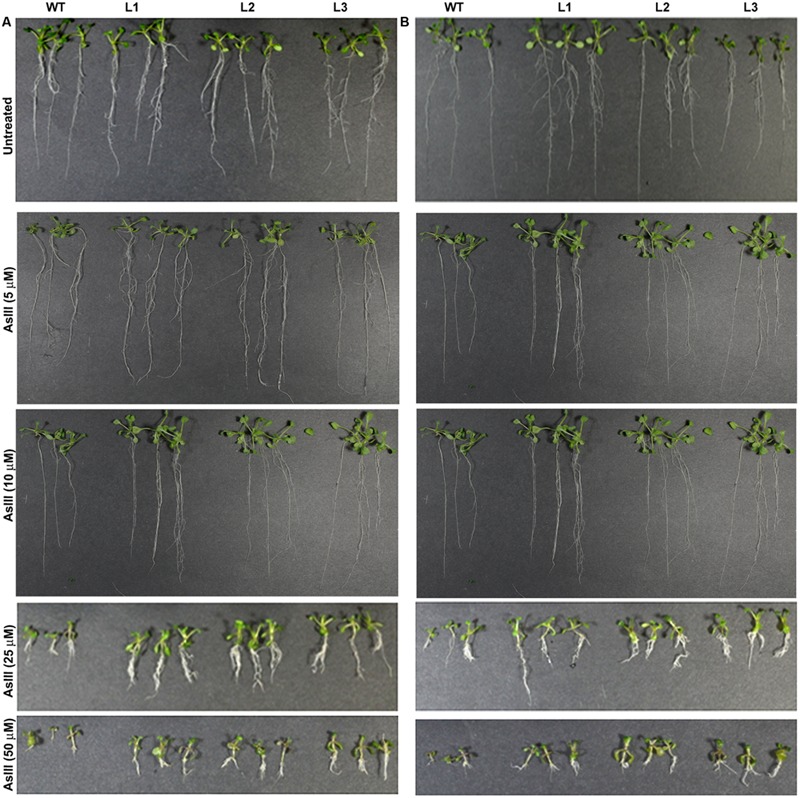
**Enhanced arsenite (AsIII) tolerance in *A. thaliana* plants transformed with **(A)***OsGrx_C7* and **(B)***OsGrx_C2.1.*** Phenotypic changes in WT and transgenic *A. thaliana* plants carrying *OsGrx_C7* and *OsGrx_C2.1* observed after grown vertically on plates of ½ × MS medium without As and with 5 μM AsIII, 10 μM AsIII, 25 μM AsIII, and 50 μM AsIII for 10 days. (*n* = 5 plants per treatment per line and repeated five times).

**FIGURE 4:**
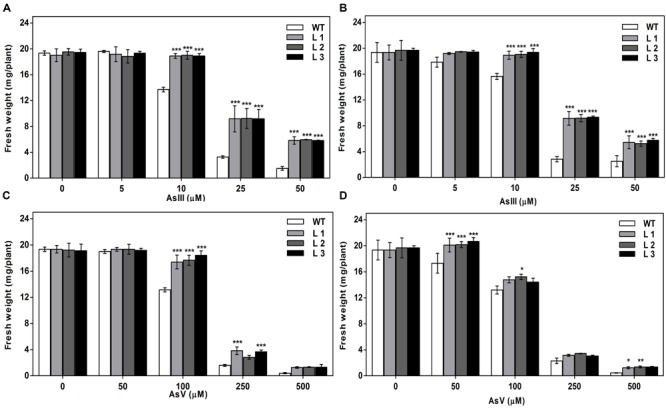
**Expression of *OsGrxs* affects the biomass accumulation during arsenic (AsIII and AsV) stress. (A)** Fresh weight of WT and *OsGrx_C7* transformed plants and **(B)** Fresh weight of WT and *OsGrx_C2.1* transformed plants during AsIII stress, **(C)** Fresh weight of WT and *OsGrx_C7* transformed plants and **(D)** Fresh weight of WT and *OsGrx_C2.1* transformed plants during AsV stress. The *A. thaliana* plants carrying *OsGrx_C7* and *OsGrx_C2.1* and WT plants were grown on plates of ½ × MS medium containing AsIII (5, 10, 25, and 50 μM) and AsV (50, 100, 250, and 500 μM) for 10 days. (*n* = 5 plants per treatment per line and repeated five times). Asterisks indicate significant differences: (^∗^*P* < 0.05; ^∗∗^*P* < 0.01; ^∗∗∗^*P* < 0.001). Error bars, mean ± SE.

**FIGURE 5:**
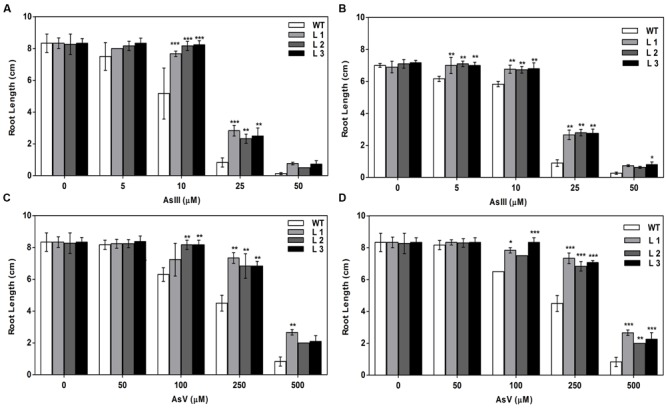
**Expression of both *OsGrxs* affects plant growth (root length) during As (AsIII and AsV) stress. (A)** Root length of WT and *OsGrx_C7* transformed plants, and **(B)** Root length of WT and *OsGrx_C2.1* transformed plants during AsIII stress, **(C)** Root length of WT and *OsGrx_C7* transformed plants and **(D)** Root length of WT and *OsGrx_C2.1* transformed plants during AsV stress. The *A. thaliana* plants carrying *OsGrx_C7* and *OsGrx_C2.1* and WT plants were grown on plates of ½ × MS medium containing AsIII (5, 10, 25, and 50 μM) and AsV (50, 100, 250, and 500 μM) for 10 days. (*n* = 5 plants per treatment per line and repeated five times). Asterisks indicate significant differences: (^∗^*P* < 0.05; ^∗∗^*P* < 0.01; ^∗∗∗^*P* < 0.001). Error bars, mean ± SE.

Germination of WT seeds were significantly inhibited on As containing medium, but *OsGrxs* expressing lines were least affected (Supplementary Figure S2A). In root growth bioassay there was no phenotypic change observed between WT and transgenic lines grown on ½ × MS medium without As. Transgenic lines were further grown in ½ × MS medium supplemented with 5 μM, 10 μM, 25 μM AsIII and 50 μM, 100 μM, 250 μM AsV. Although, WT as well as transgenic lines, showed root growth inhibition in the presence of the AsV and AsIII in a concentration-dependent manner (**Figures 2A,B** and **3A,B**). *OsGrxs* overexpressing lines showed less root growth inhibition compared to WT, which was more significant at 25 μM AsIII and 250 μM AsV concentrations. The tolerance was also visible in aerial tissues, as transgenic lines grew better and attained significantly higher fresh weight as compared to WT (**Figure 4**). Root hair growth and elongation severely inhibited in WT plants as compared to transgenic lines under AsIII toxicity (**Figure 5**; Supplementary Figure S2B).

**FIGURE 6:**
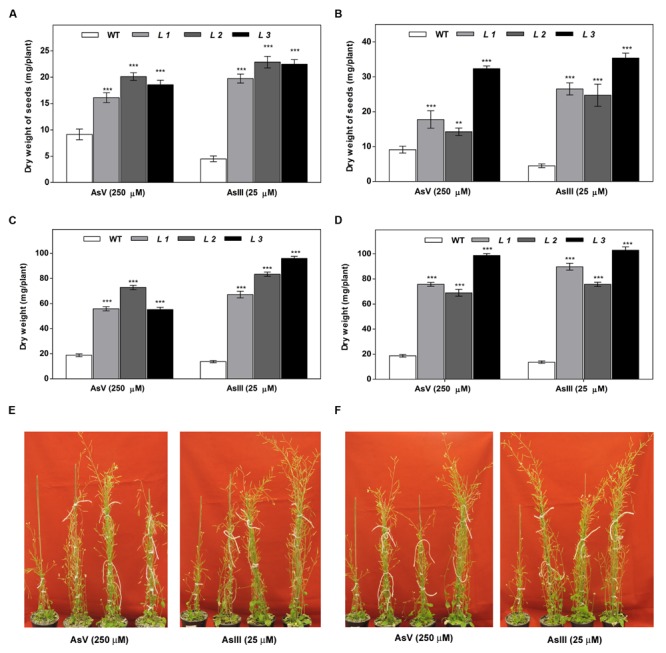
**Higher biomass accumulation in above-ground tissues of transgenic *A. thaliana* plants after long-term cultivation in soil under As (AsIII and AsV) stress. (A)** The dry weight of seeds of WT and *OsGrx_C7* transformed plants, **(B)** Dry weight of seeds of WT and *OsGrx_C2.1* transformed plants, **(C)** Dry weight of WT and *OsGrx_C7* transformed plants, **(D)** Dry weight of WT and *OsGrx_C2.1* transformed plants. The *A. thaliana* plants carrying **(E)** *OsGrx_C7*, **(F)** *OsGrx_C2.1* and WT plants grown in soil spiked with 250 μM As(V) and 25 μM As(III) for 45 days (*n* = 8 plants per treatment per line and repeated three times). Asterisks indicate significant differences: (^∗∗^*P* < 0.01; ^∗∗∗^*P* < 0.001). Error bars, mean ± SE.

The As response of the transgenic lines was examined in 6 weeks old plants. For this, 12-day-old seedlings were transferred to pots and allowed to grow for two more weeks. Then, the plants were irrigated with or without sodium arsenate (250 μM) and sodium arsenite (25 μM) in Hoagland nutrient solution for 6 weeks. The phenotypic effects of As treatments after 6 weeks are shown in **Figure 6**. Both AsIII and AsV resulted in retardation of seedling development in WT seedlings. The transgenic seedlings showed less severe symptoms and continued to grow. Fresh biomass, dry weight and seeds from these plants grown on As for 6 weeks revealed higher biomass and seeds yield in transgenic lines compared to WT plants grown on AsIII and AsV. These results indicate that transgenic plants displayed improved tolerance to As stress.

### Overexpression of *OsGrx*s reduces As Accumulation in *A. thaliana*

**FIGURE 7:**
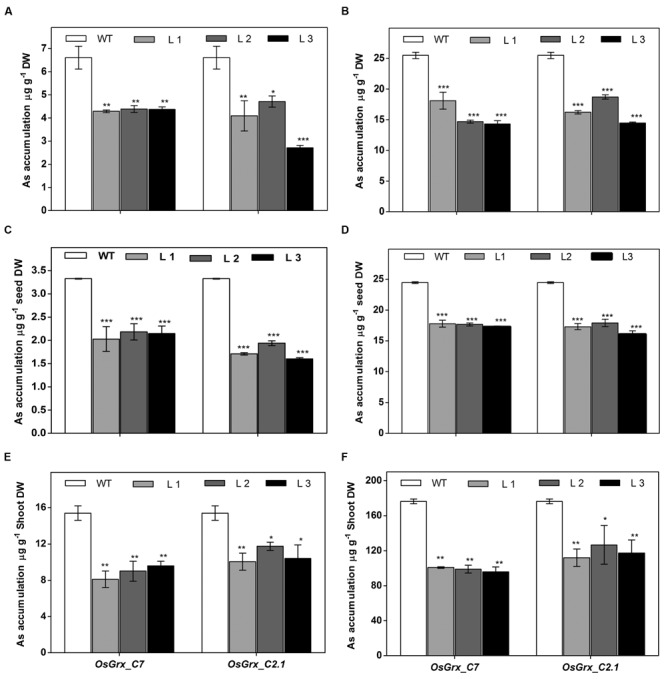
**Heterologous expression of *OsGrxs* displayed significantly reduced arsenic accumulation in *A. thaliana*.** Arsenic accumulation in plants grown in hydroponics with **(A)** 25 μM AsIII, **(B)** 250 μM AsIII for 7 days and whole plant was digested and subjected to ICPMS analysis (*n* = 10 plants per treatment per line and repeated three times). Arsenic accumulation in seeds when plants are grown in soil spiked with **(C)** 25 μM AsIII, **(D)** 250 μM AsV for 45 days. Arsenic accumulation in rosette leaves + stalks when plants are grown in soil spiked with **(E)** 25 μM AsIII and **(F)** 250 μM AsV for 45 days (*n* = 8 plants per treatment per line and repeated three times). Asterisks indicate significant differences: (^∗^*P* < 0.05; ^∗∗^*P* < 0.01; ^∗∗∗^*P* < 0.001). Error bars, mean ± SE.

The *OsGrx*s expressing transgenic plants grown in hydroponics medium containing AsIII and AsV. The whole plant was digested using concentrated HNO_3_ for ICP-MS analysis. The results of ICP-MS analysis displayed significantly less As accumulation in the transgenic plants (**Figures 7A,B**). Arsenic accumulation in seeds and shoot tissues were also measured after long-term exposure of AsIII and AsV in soilrite irrigated with As-contaminated water (described in above section). ICP-MS analysis showed *ca.* 38% reduced As accumulation in seeds under AsV (**Figure 7C**) and AsIII stress (**Figure 7D**) compared to WT. Whereas, in shoot tissues (Shoot + rosette leaves) *ca.* 40% less As accumulated after exposure to AsV (**Figure 7E**) and AsIII (**Figure 7F**) compared to WT. A decrease in As content in seeds and shoot tissues of *OsGrxs* expressing transgenic lines confirms the role of *OsGrx*s in lower As uptake and higher AsIII extrusion.

### Altered Expression of As Transporters in *OsGrx*s Expressing *A. thaliana* Transgenic Lines

**FIGURE 8:**
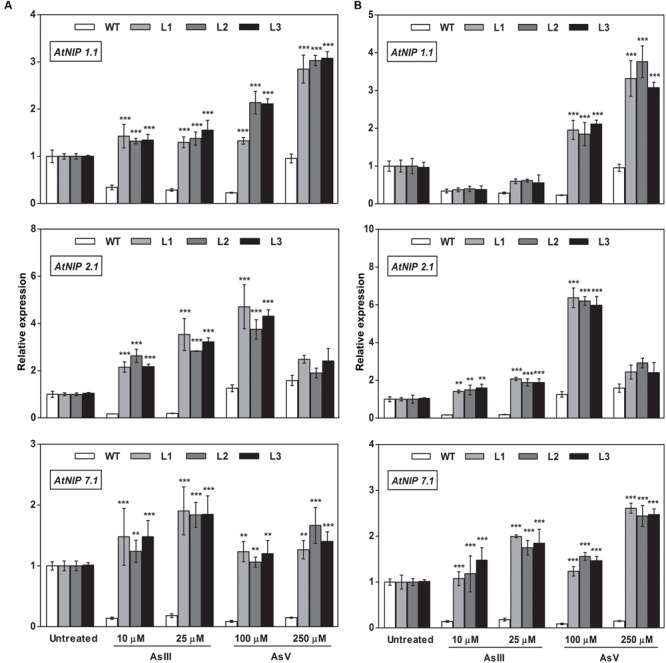
**Heterologous expression of *OsGrxs* increases the mRNA level of *A. thaliana* aquaporins (*AtNIP1.1; AtNIP2.1; AtNIP7.1*) in the shoot.** Transgenic *A. thaliana* plants carrying **(A)** *OsGrx_C7* and **(B)** *OsGrx_C2.1* were grown on ½ × MS medium and transferred to hydroponic medium supplemented with 10 μM AsIII, 25 μM AsIII and 100 μM AsV, 250 μM AsV, grown for 1 week. Samples were harvested and washed thoroughly with RNase-free water to remove As attached on the plant surface. Total RNA of *A. thaliana* shoot was extracted for qRT-PCR analysis. Asterisks indicate significant differences: (^∗∗^*P* < 0.01; ^∗∗∗^*P* < 0.001). Error bars, mean ± SE.

**FIGURE 9:**
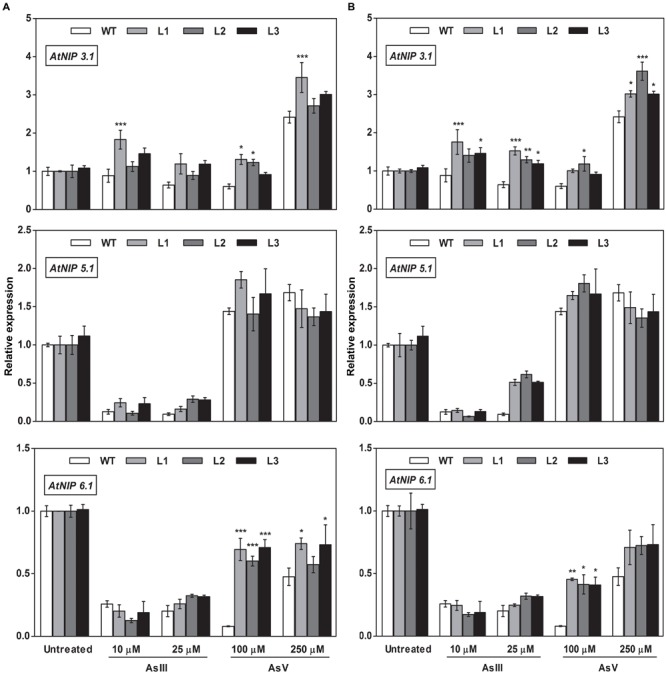
**Overexpression of *OsGrxs in A. thaliana* alters the mRNA level of aquaporins (*AtNIP3.1; AtNIP5.1; AtNIP 6.1*) in shoot tissues.** Transgenic *A. thaliana* plants carrying **(A)** *OsGrx_C7* and **(B)** *OsGrx_C2.1* were grown on ½ × MS medium and transferred to hydroponic medium supplemented with 10 μM AsIII, 25 μM AsIII and 100 μM AsV, 250 μM AsV, grown for 1 week. Samples were harvested and washed thoroughly with RNase-free water to remove As attached on the plant surface. Total RNA of *A. thaliana* shoot was extracted for qRT-PCR analysis. Relative expressions of *AtNIPs* (*AtNIP3.1; AtNIP5.1; AtNIP6.1*) were represented. Asterisks indicate significant differences: (^∗^*P* < 0.05; ^∗∗^*P* < 0.01; ^∗∗∗^*P* < 0.001). Error bars, mean ± SE.

**FIGURE 10:**
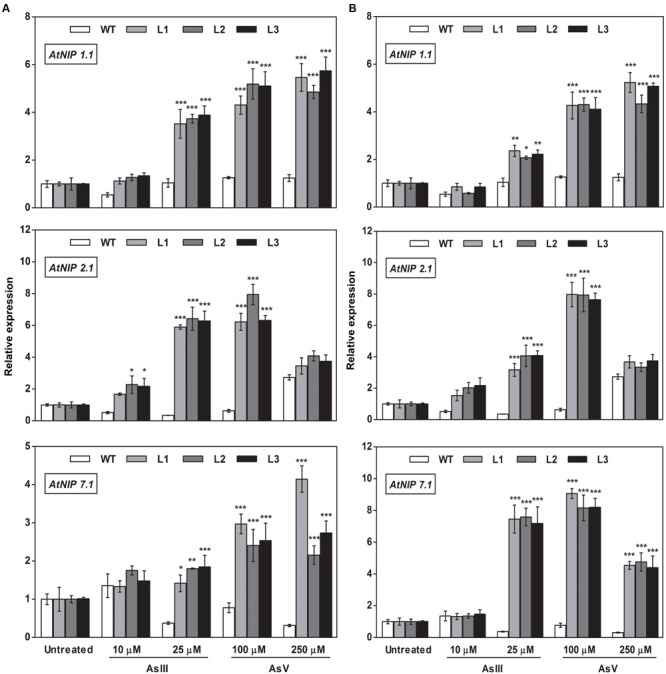
**Overexpression of *OsGrxs* in *A. thaliana* increases the mRNA level of aquaporins (*AtNIP1.1; AtNIP2.1; AtNIP7.1*) in the root.** Transgenic *A. thaliana* plants carrying **(A)** *OsGrx_C7* and **(B)** *OsGrx_C2.1* were grown on ½ × MS medium and transferred to hydroponic medium supplemented with 10 μM AsIII, 25 μM AsIII and 100 μM AsV, 250 μM AsV, grown for 1 week. Samples were harvested and washed thoroughly with RNase-free water to remove As attached on the plant surface. Total RNA extracted from roots of *A. thaliana* for qRT-PCR analysis. Relative expressions of *AtNIP*s (*AtNIP1.1; AtNIP2.1; AtNIP7.1*) were represented. Asterisks indicate significant differences: (^∗^*P* < 0.05; ^∗∗^*P* < 0.01; ^∗∗∗^*P* < 0.001). Error bars, mean ± SE.

**FIGURE 11:**
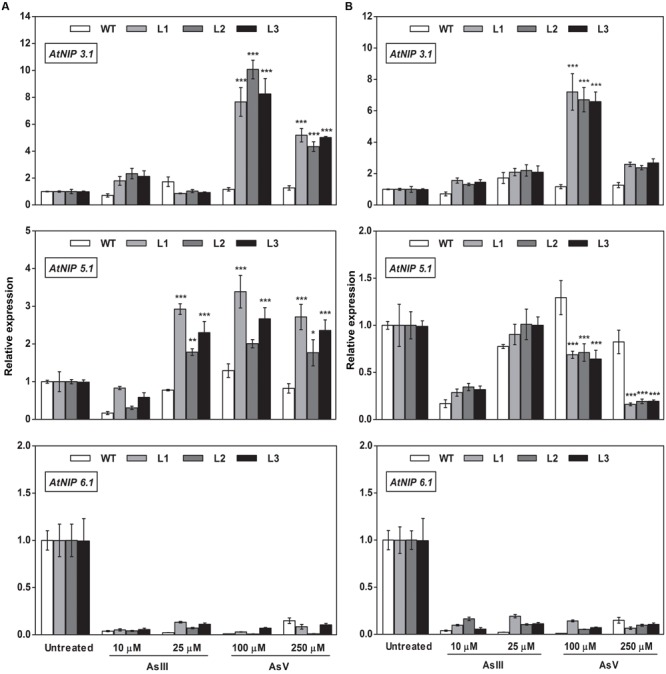
**Overexpression of *OsGrxs* alters the mRNA level of *A. thaliana* aquaporins (*AtNIP3.1; AtNIP5.1; AtNIP 6.1*) in the root.** Transgenic *A. thaliana* plants carrying **(A)** *OsGrx_C7* and **(B)** *OsGrx_C2.1* were grown on ½ × MS medium and transferred to hydroponic medium supplemented with 10 μM AsIII, 25 μM AsIII and 100 μM AsV, 250 μM AsV, grown for 1 week. Samples were harvested and washed thoroughly with RNase-free water to remove As attached on the plant surface. Total RNA was extracted from roots of *A. thaliana* for qRT-PCR analysis. Relative expressions of *AtNIPs* (*AtNIP3.1; AtNIP5.1; AtNIP6.1*) were represented. Asterisks indicate significant differences: (^∗^*P* < 0.05; ^∗∗^*P* < 0.01; ^∗∗∗^*P* < 0.001). Error bars, mean ± SE.

To elucidate the rationale behind tolerance of transgenic lines to AsIII and AsV, the expression of aquaporin’s subfamily noduline-26 like intrinsic proteins (*NIP*), involved in AsIII transport in plants, were also examined. *OsGrxs* expressing lines displayed enhanced expression of *AtNIP1;1, AtNIP2;1, AtNIP3;*1, and *AtNIP7;1* during AsV stress in both shoot (**Figures 8** and **9**) and root (**Figures 10** and **11**). However, *AtNIP6;*1 was slightly upregulated only in shoots under AsV stress while, *AtNIP5;*1 showed downregulation in roots of *OsGrx_C2.1* expressing transgenic *A. thaliana*. It has been already reported that plant NIPs are involved in translocation of As(OH)_3_ against a concentration gradient (Bienert et al., 2008). AsV, being a phosphate analog, is taken up in plants via phosphate transporter and get reduced to AsIII, which might create concentration gradient for AsIII and transported by NIPs. Increased expression of NIPs in transgenic root indicated that they might involve in the extrusion of As(OH)_3_ in medium (**Figure 10**).

During AsIII stress *AtNIPs* have different expression pattern, *OsGrx_C7* and *OsGrx_C2.1* expressing lines displayed enhanced expression of *AtNIP1;1, AtNIP2;1 AtNIP7;1* in both root and shoot. During AsIII exposure, NIPs were upregulated to transport and extrude AsIII in the medium. The concentration gradient might be created by depletion of AsIII in medium (uptake and sequestered in plant vacuole) or by extracellular GSH binds to AsIII. Also, transgenic lines displayed unaltered expression of *AtNIP3.1, AtNIP6.1* in *OsGrx_C7* and *OsGrx_C2.1* expressing lines during AsIII stress. *AtNIP5.1* showed higher expression in *OsGrx_C7* transgenic root. However, in untreated condition expression levels of mRNA transcripts for these genes are least affected in transgenic lines. The altered transcript level of aquaporin in the presence of *OsGrx*s under AsV and AsIII stress confirmed that both *OsGrx*s alter the expression of aquaporin for As tolerance and AsIII extrusion.

### The Oxidoreductase Activity of *OsGrx*s Increases after As Treatment

**FIGURE 12:**
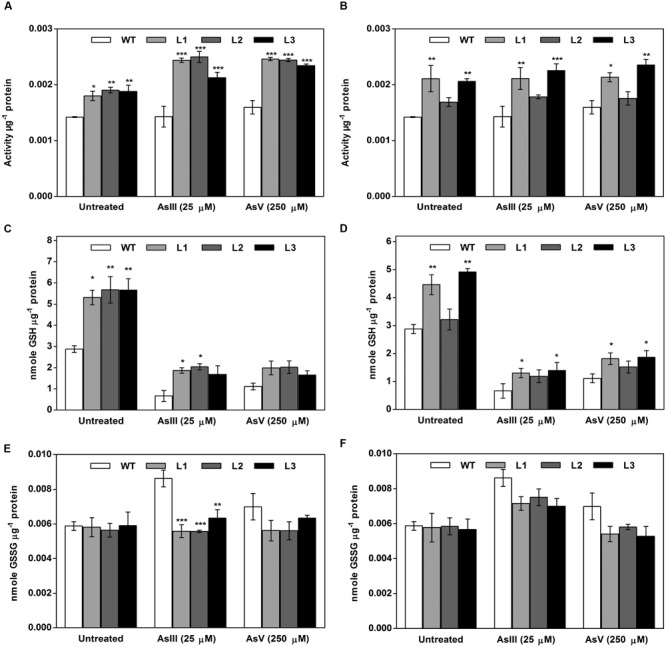
**Both *OsGrx*s displayed enhanced Grx activity and intracellular GSH content during As stress in transgenic *A. thaliana* compared to WT. (A)** Enhanced Grx activity in *OsGrx_C7* transformed plants, **(B)** enhanced Grx activity in *OsGrx_C2.1* transformed plants. **(C)** Intracellular GSH content in *OsGrx_C7* transformed plants, **(D)** intracellular GSH in *OsGrx_C2.1* transformed plants, **(E)** intracellular GSSG content in *OsGrx_C7* transformed plants, **(F)** intracellular GSSG in *OsGrx_C2.1* transformed plants. Transgenic *A. thaliana* plants were grown on ½ × MS medium and transferred to hydroponic medium supplemented with 25 μM AsIII and 250 μM AsV, grown for 1 week. Samples were harvested and washed thoroughly with water to remove As attached on the plant surface. The plants were homogenized in potassium phosphate buffer pH 7.8 (100 mM, ice-cold) with 5% 5-sulfosalicylic acid and used to determine intracellular glutathione (oxidized and reduced). The reaction started by adding 0.2 U of GR and the kinetics of DTNB conversion to TNB were followed spectrophotometrically at 412 nm. GSH concentrations calculated from standard curves obtained with various GSH and GSSG concentrations. Asterisks indicate significant differences: (^∗^*P* < 0.05; ^∗∗^*P* < 0.01; ^∗∗∗^*P* < 0.001). Error bars, mean ± SE.

To test whether, As tolerance in transgenic *A. thaliana* was correlated to *OsGrx*s activity, glutaredoxin activity was measured in, soluble fractions of leaf protein from plants exposed to AsIII (25 μM) and AsV (250 μM). *OsGrx_C7* expressing transgenic lines had *c.a* 27%, and *OsGrx_C2.1* expressing transgenic lines had *c.a*. 30% Grx-specific activity compared with WT in the control condition. On exposure of As, glutaredoxin activity in *OsGrx_C7* expressing transgenic lines was increased up to *c.a* 40% and *c.a* 35% compared to WT in AsIII and AsV stress, respectively. Moreover, *OsGrx_C2.1* expressing transgenic lines showed increased glutaredoxin activity up to *c.a* 35% and *c.a* 30% compared to WT in AsIII and AsV stress respectively (**Figures 12A,B**).

### Heterologous Expression of *OsGrxs* Maintains the Cellular Glutathione Level under As Stress

*In planta* expression of *OsGrxs* displayed high GSH content compared to WT plants. Under AsIII stress, intracellular GSH concentration was increased up to 65% in *OsGrx_C7* expressing plants and up to 45% in *OsGrx_C2.1* expressing plants compared to WT. While on AsV exposure, intracellular GSH concentration was increased *c.a* 35% and *c.a* 30% in plants expressing *OsGrx_C7* and *OsGrx_C2.1* respectively compared to WT (**Figures 12C,D**). Expression of *OsGrxs* displayed decreases in GSSG content compared to WT plants (**Figures 12E,F**).

## Discussion

Members of Grx protein family in rice, *A. thaliana* as well other higher plants reported to involve in reversible glutathionylation and iron metabolism (Lillig et al., 2008). The involvement of *OsGrxs* in As transport is still unexplored. Here, we described two rice glutaredoxins (*OsGrx_C7* and *OsGrx_C2.1*) had a significant role in As tolerance. Results showed that transgenic expression of both *OsGrxs* in *A. thaliana* was sufficient to increase plant tolerance to As. Earlier studies reported that expression of *OsGrx_C7* and *OsGrx_C2.1* was induced during As stress in rice in addition to defense and stress responsive genes, transporters, heat-shock proteins, metallothioneins, sulfate-metabolizing proteins (Norton et al., 2008; Chakrabarty et al., 2009; Gautam et al., 2012; Nguyen et al., 2014). In this study, similar results were observed through RT-PCR analysis, upon AsIII and AsV exposure (**Figure 1**) supporting the possible involvement of *OsGrxs* in As metabolism and transport. These results suggested that on As exposure both *OsGrxs* show upregulation to cope with redox imbalance created by arsenic, as heavy metals such as arsenate, arsenite, chromium, cadmium, leads to increasing oxidative stress in cells which create redox imbalance and cellular damage (Stohs and Bagchi, 1995). The expression of both *OsGrxs* was higher in sensitive varieties due to higher As uptake and translocation compared to tolerant variety. These results also supported by previous studies of our group where sensitive rice variety showed higher upregulation of glutaredoxins and genes involved in thiol metabolism due to greater As influx (13-fold) than tolerant genotype at 25-μM AsIII exposure in hydroponics (Dave et al., 2013). In contrast to sensitive variety, tolerant genotype displayed relatively low expression of glutaredoxins, genes involved in thiol metabolism, and less phytochelatin accumulation that was enough to detoxify the low levels of As. However, phytochelatin accumulation was also greater in the sensitive varieties, but it was not adequate to detoxify the high As content, and therefore it leads to strong up-regulation of the antioxidant gene including As responsive glutaredoxins (Dave et al., 2013).

In the present study, we chose to utilize a heterologous transgenic expression method to evaluate the function of *OsGrxs* in plants and demonstrated that overexpression of any one of two As-induced *OsGrxs* genes can confer As resistance in plants (**Figures 2** and **3**). The differences in relative root growth were significant and reproducible (**Figure 4**). Furthermore, lines expressing these two genes showed lower As content in whole plants (hydroponics), shoots (leaves + shoot) and seeds of transgenic *A. thaliana* (**Figure 7**). Sundaram et al. (2009) also reported a similar observation, where a *Grx* from *Pteris vittata* (*PvGRX5*) expressed in *A. thaliana*, resulted in more As tolerance compared with control lines based on germination, root growth, and whole plant growth. *PvGRX5* expressing lines also contained significantly lower total As compared to control lines following treatment with AsV. However, they have failed to identify how *PvGRX5* affects AsV uptake, AsIII translocation and eﬄux.

Based on our earlier work on yeast (Verma et al., 2016), we raised the question whether the As uptake and eﬄux regulated by *OsGrx*s affected the transcript level of aquaporins and other As-related transporters. In the present study, we demonstrated that *A. thaliana NIP*s including *AtNIP1;1, AtNIP2;1* and *AtNIP7;1* were upregulated during AsV stress (**Figures 8** and **10**). These NIPs are known to involve in AsIII uptake and translocation. Thus, confirmed that both *OsGrxs* were involved in AsV tolerance by regulating the transcript level of aquaporins. Both *OsGrxs* are also involved in AsV reduction (Verma et al., 2016) and might create AsIII gradient to extrude via aquaporins down to concentration gradient. In previous reports, it was demonstrated that aquaporins transported AsIII in the form of As(OH)_3_ that are not transported by active transporters such as *ABCC* (plants) and *Ycf1, Acr3* (Yeast) (Wysocki et al., 1997). In our study, we also found that *OsGrx_C7* and *OsGrx_C2.1* expressing lines displayed enhanced expression of *AtNIP1;1, AtNIP2;1* and *AtNIP7;1* during AsIII stresses (**Figures 8** and **10**). It has been reported that the expression of several *A. thaliana* NIPs, *AtNIP5;1, AtNIP6;1*, and *AtNIP7;1*, enhances the ability of yeast cells to extrude AsIII (Bienert et al., 2008; Isayenkov and Maathuis, 2008). Earlier it was also known that rice NIP aquaporin *OsNIP2;1* (Lsi1) is also able to eﬄux AsIII out of rice root cells into the external medium (Zhao et al., 2010). Whereas, suppressing the expression of *OsNIP2;1* in rice and *AtNIP1;1*, and *AtNIP7;1* in *A. thaliana* resulted in the decreased uptake of AsIII (Isayenkov and Maathuis, 2008; Ma et al., 2008; Kamiya et al., 2009). These studies suggested that NIPs facilitate the bidirectional movement of AsIII in plants. Recently Xu et al. (2015) reported that *AtNIP3;1* is involved in AsIII uptake and root-to-shoot translocation in *A. thaliana*, probably as a passive and bidirectional AsIII transporter. Here, the expression of *AtNIP3;1* was not as high as compared with other NIPs during AsIII stress (**Figures 9** and **11**). We also observed tissue-specific as well as *Grx* specific modulation of few NIPs. Thus, our results suggest that overexpression of rice *Grxs* modulates the expression of different NIPs in a tissue-specific manner and some of the NIPs might involve in the extrusion of AsIII from the roots of *A. thaliana* upon exposure to AsIII and AsV stresses. Moreover, we have searched the different data sets provided by various authors, in the data set of Chakrabarty et al. (2009), we observed the co-expression of the glutaredoxins [Os01g27140 (*OsGrx_C7*) and Os02g40500 (*OsGrx_C2.1*)] with the aquaporins in Rice genome array (Supplementary Figure S3). These results support our data that overexpression of *OsGrxs* alters the expression pattern of certain aquaporins (NIPs) for AsIII extrusion.

The As detoxification involves transport of As(GS)_3_ complex from the cytosol to the extracellular medium and or vacuolar lumen by active transporters. GSH might also be exported into the extracellular medium under As stress (Thorsen et al., 2012), lead to GSH depletion in the cell cytosol (Meharg and Hartley-Whitaker, 2002; Tripathi et al., 2007, 2012). Measurement of total GSH and GSSG during As stress gave the evidence of the involvement of both *OsGrxs* in maintaining of cellular GSH content (**Figure 12**). Thus, both *OsGrxs* maintain cellular redox status either by increasing GSH production or by GSH recycling. *Grxs* are also known to involve in defense activities by controlling the expression of antioxidant genes. *Grxs* able to catalyzed reversible glutathionylation of protein during adverse condition to protect cellular proteins damage by ROS (Klatt and Lamas, 2000). *Grxs* act by interacting with target proteins, altering their redox states and function. *Grxs* also control the expression of several other genes by regulating the DNA-binding activities of redox-sensitive transcription factors such as *AP-1, NF-kβ*, and *OxyR* (Hirota et al., 1997; Luikenhuis et al., 1998; Zheng et al., 1998; Harper et al., 2001). Here, we propose that both *OsGrxs* maintain the redox status of the cells and modulates the expression of *AtNIPs* under As stress, which might be accounted for the extrusion of intracellular AsIII and minimize the translocation of toxic As to edible parts of plants (**Figure 7**). Many others researchers had altered As tolerance in transgenic plants via over-expressing specific genes encoding enzymes involved in the synthesis of phytochelatins in heavy metal detoxification (Dhankher et al., 2002; Gasic and Korban, 2007). However, the characterization of *OsGrxs* represents the first successful attempt toward a genetic dissection of AsIII tolerance mechanism in the rice and is likely independent of phytochelatins and possibly involves aquaporins. Thus, our results strongly suggest that *OsGrx*s could be used to engineer transgenic crops with an ability to accumulate low As levels in seeds and shoot parts.

It is the first report on identification and characterization of a member of Grx family protein, in rice involved in As transport. These *OsGrxs* participate in reducing AsIII uptake and AsIII extrusion by altering the aquaporins (*AtNIPs*) transcripts in *A. thaliana* under As stress. *OsGrxs* also play a crucial role in maintaining the intracellular GSH pool and redox status of the cells under As stress, indicating that the thiol/disulfide couple is ideally suited to redox modulation, serving as redox sensor and a switch to alter protein structures and activities leading to modulation of several gene functions. Although the mechanistic details are not entirely clear, it is likely that there are several independent and interacting genes are involved. Further analysis will help to decipher the exact mechanism involved in *OsGrxs* mediated As metabolism in plants. However, the observations of this study are of central importance for the development of rice genotypes with less As accumulation in their edible parts through upregulation of *OsGrxs* to meet the perspective of As safe rice.

## Author Contributions

DC, SM, and PV conceived and designed the experiments. All experiments performed by PV and SV. DC, SM, and PV analyzed the data. DC, RT, VP, and OD revised the paper. DC, PV, RT, OD, and SV wrote the paper. All authors have read and approve of the final manuscript.

## Conflict of Interest Statement

The authors declare that the research was conducted in the absence of any commercial or financial relationships that could be construed as a potential conflict of interest.
