# Corrigendum: Overexpression of Rice Glutaredoxin *OsGrx_C7* and *OsGrx_C2.1* Reduces Intracellular Arsenic Accumulation and Increases Tolerance in *Arabidopsis thaliana*

**DOI:** 10.3389/fpls.2016.01048

**Published:** 2016-07-12

**Authors:** Pankaj K. Verma, Shikha Verma, Veena Pande, Shekhar Mallick, Rudra Deo Tripathi, Om P. Dhankher, Debasis Chakrabarty

**Affiliations:** ^1^Genetics and Molecular Biology Division, Council of Scientific and Industrial Research-National Botanical Research InstituteLucknow, India; ^2^Department of Biotechnology, Kumaun UniversityNainital, India; ^3^Environmental Biotechnology Division, Council of Scientific and Industrial Research-National Botanical Research InstituteLucknow, India; ^4^Stockbridge School of Agriculture, University of MassachusettsAmherst, Massachusetts

**Keywords:** arsenic, GSH, *OsGrxs*, glutaredoxin, *Oryza sativa*, aquaporin

Reason for Corrigendum:

There was a mistake in the first section of the Results. LOC_Os01g40500 should be LOC_Os02g40500. The authors apologize for the mistake. This error does not change the scientific conclusions of the article in any way.

## Author contributions

DC, SM, and PV conceived and designed the experiments. All experiments performed by PV and SV. DC, SM, and PV analyzed the data. DC, RD, VP, and OD revised the paper. DC, PV, RD, OD, and SV wrote the paper. All authors have read and approve of the final manuscript.

### Conflict of interest statement

The authors declare that the research was conducted in the absence of any commercial or financial relationships that could be construed as a potential conflict of interest.

