# Corrigendum (2): Overexpression of Rice Glutaredoxin *OsGrx_C7* and *OsGrx_C2.1* Reduces Intracellular Arsenic Accumulation and Increases Tolerance in *Arabidopsis thaliana*

**DOI:** 10.3389/fpls.2017.01884

**Published:** 2017-11-03

**Authors:** Pankaj K. Verma, Shikha Verma, Veena Pande, Shekhar Mallick, Rudra Deo Tripathi, Om P. Dhankher, Debasis Chakrabarty

**Affiliations:** ^1^Genetics and Molecular Biology Division, Council of Scientific and Industrial Research-National Botanical Research Institute, Lucknow, India; ^2^Department of Biotechnology, Kumaun University, Nainital, India; ^3^Environmental Biotechnology Division, Council of Scientific and Industrial Research-National Botanical Research Institute, Lucknow, India; ^4^Stockbridge School of Agriculture, University of Massachusetts, Amherst, MA, United States

**Keywords:** arsenic, GSH, *OsGrxs*, glutaredoxin, *Oryza sativa*, aquaporin

There was a mistake in the Figure 3 as published. During the preparation of Figure 3 in a photo editor software, we unintentionally duplicated the 10 μM AsIII figures. The correct version of Figure 3 appears below. The authors apologize for the mistake. This error does not change the scientific conclusions of the article in any way.

**Figure 3:**
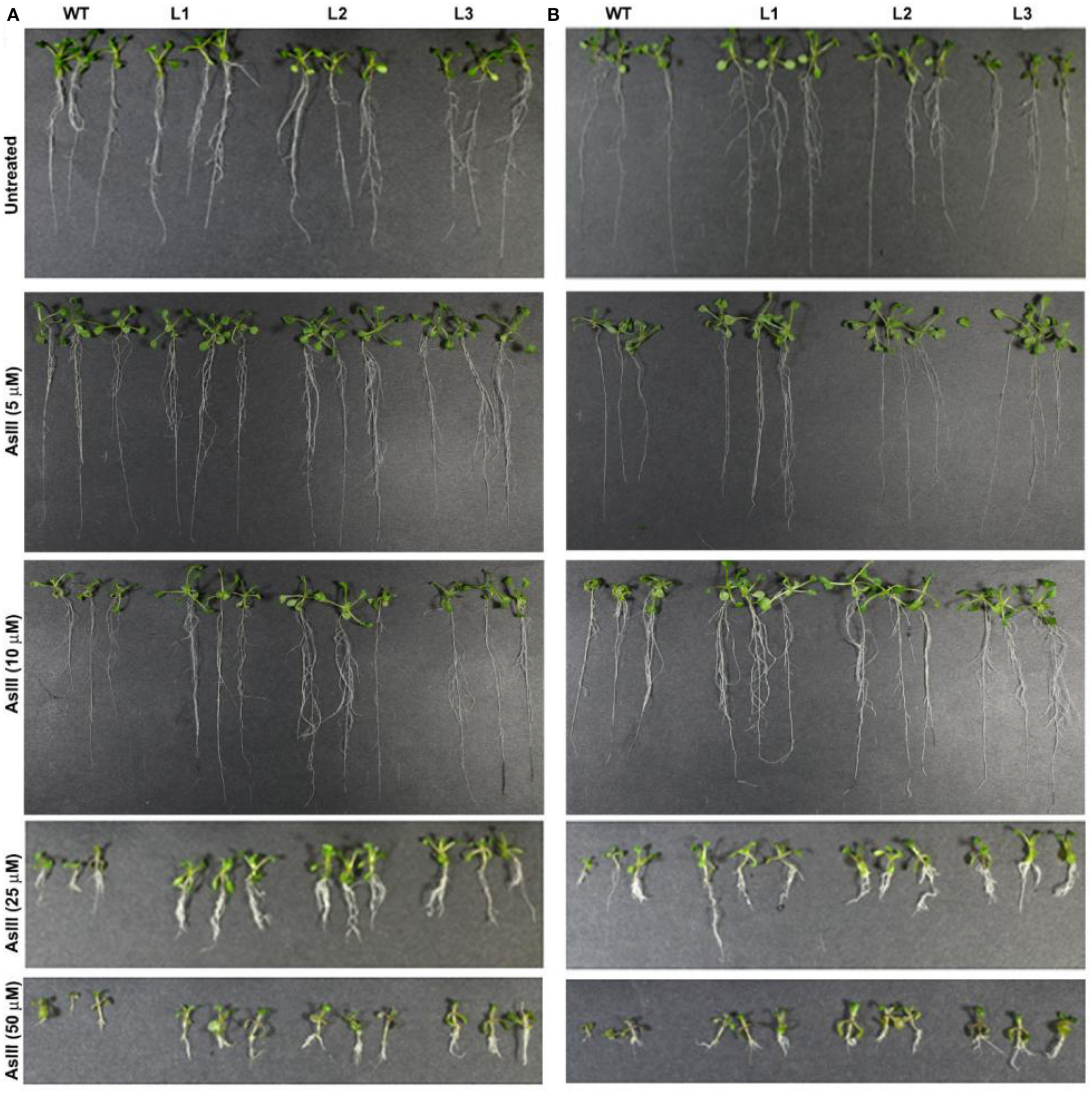
Enhanced arsenite (AsIII) tolerance in *A. thaliana* plants transformed with **(A)** *OsGrx_C7* and **(B)** *OsGrx_C2.1*. Phenotypic changes in WT and transgenic *A. thaliana* plants carrying *OsGrx_C7* and *OsGrx_C2.1* observed after grown vertically on plates of ½ × MS medium without As and with 5 μM AsIII, 10 μM AsIII, 25 μM AsIII, and 50 μM AsIII for 10 days (*n* = 5 plants per treatment per line and repeated five times).

## Conflict of interest statement

The authors declare that the research was conducted in the absence of any commercial or financial relationships that could be construed as a potential conflict of interest.

