# Supplementary material for: Overexpression of Rice Glutaredoxin OsGrx_C7 and OsGrx_C2.1 Reduces Intracellular Arsenic Accumulation and Increases Tolerance in Arabidopsis thaliana
Source: Front Plant Sci. 2016 Jun 1;7:740. doi: 10.3389/fpls.2016.00740 (PMC4887470; doi:10.3389/fpls.2016.00740)
Supplement: Supplementary file 1 [file Data_Sheet_1.PDF]

Supplementary Material

Overexpression of rice glutaredoxin *OsGrx_C7* and *OsGrx_C2.1* reduces intracellular arsenic accumulation and increases tolerance in *Arabidopsis thaliana*

Pankaj Kumar Verma[1,3], Shikha Verma[1,3], Veena Pande[3], Shekhar Mallick[2], Rudra Deo Tripathi[2], Om Parkash Dhankher[4], Debasis Chakrabarty[1*]

*** Correspondence:** Corresponding Author: chakrabartyd@nbri.res.in

Supplementary Figures

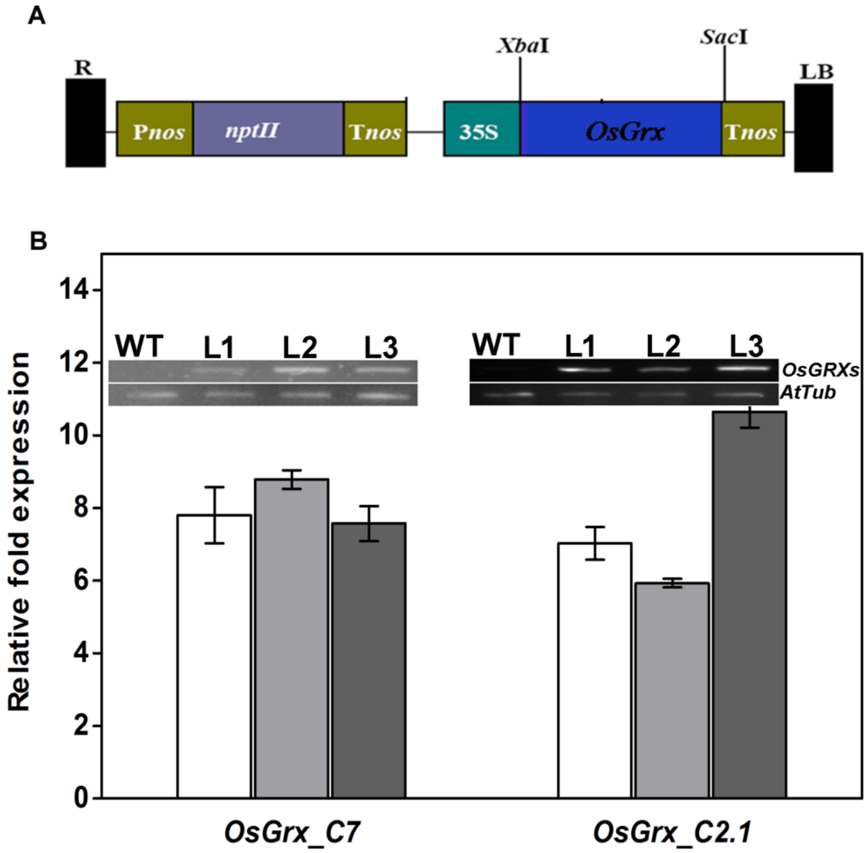

Supplementary Figure 1. Expression of *OsGrxs* in transgenic *A. thaliana*. (A) Physical map of pBI121-*OsGrxs* genes under control of the 35S promoter, (B) *OsGrxs* transcript levels were measured by qRT-PCR. Inset: Semi-quantitative RT-PCR analysis of two-week-old WT and independent *OsGrxs* transgenic lines.

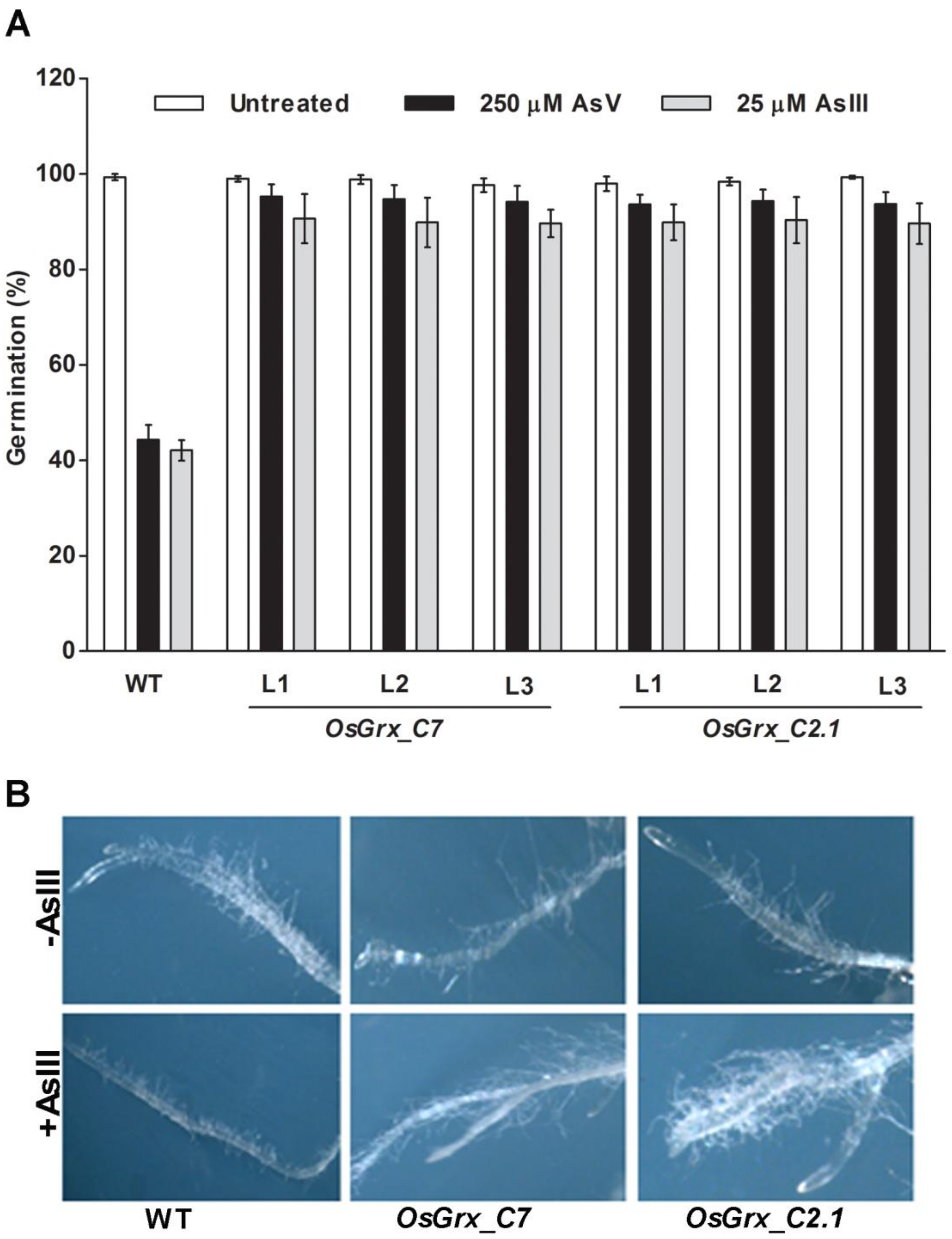

Supplementary Figure2. Arsenic tolerance of *A. thaliana* seedlings expressing *OsGrxs*. (A) Germination of WT and transgenic *A. thaliana* lines on ½x MS medium in absence or presence of 250 µM AsV and 25 µM AsIII, (B) root hair growth in WT and transgenic lines after grown in plates of ½x MS medium containing 25 µM AsIII for ten days (n = 5 plants per treatment per line and repeated 5 times). Error bars, mean ± SE.

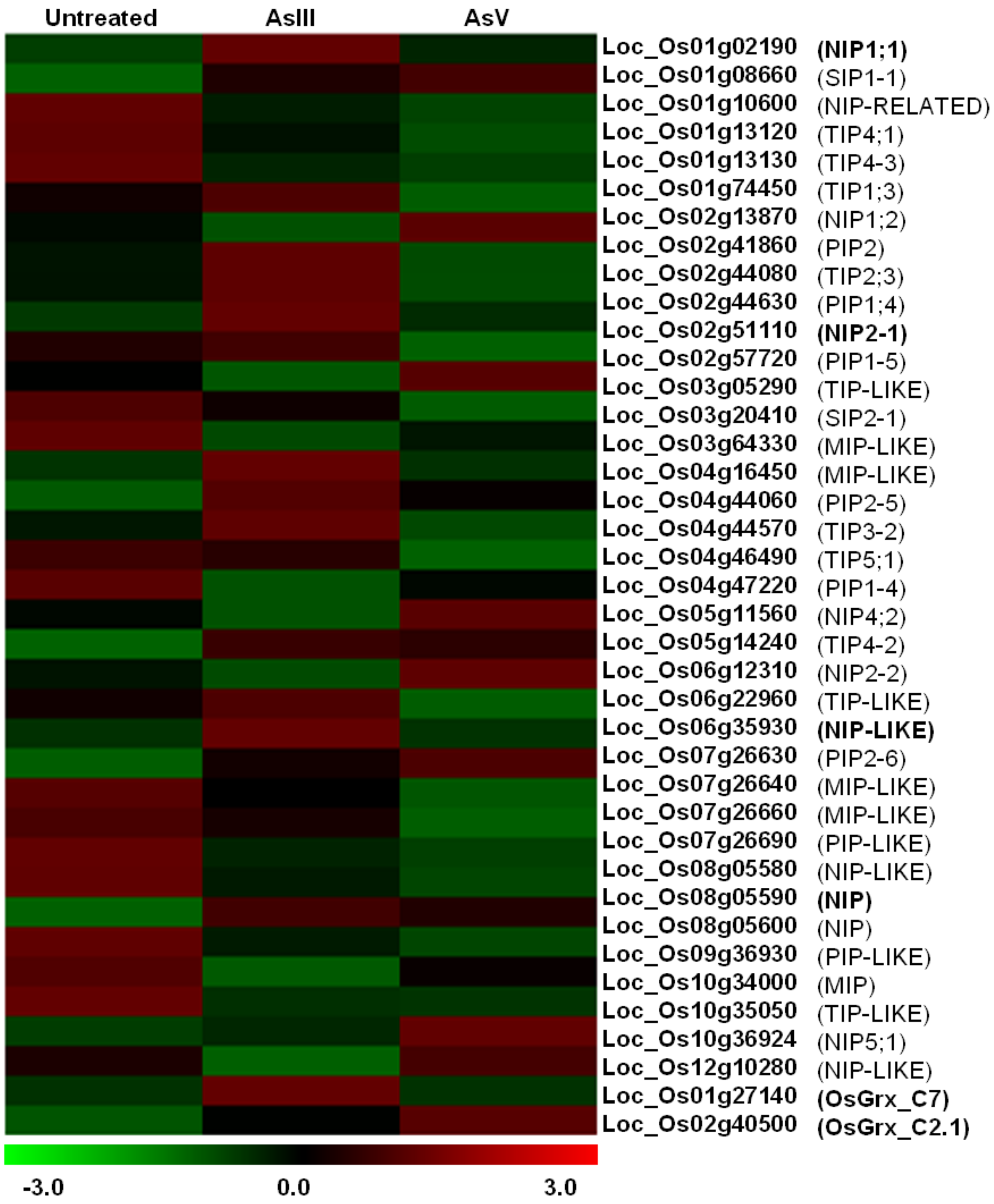

Supplementary Figure3. Expression pattern of rice aquaporin and glutaredoxin (*OsGrxs*) during AsV (250 µM), AsIII (25 µM) stress, displayed upregulation of aquaporin family proteins along with glutaredoxins (OsGrx_C7 and OsGrx_C2.1).

Supplementary Tables

Supplementary Table1. The gene primers used in study

**Primers**	**Sequences (5’-3’)**
OsGrx_C7 **F**	CAAGAAGAAAATGGACAGGGTGAAC
OsGrx_C7 **R**	TCGTTGGTGCTACACCCAGAG
OsGrx_C2.1 **F**	AGAAGGGATTCGTCAGTCGG
OsGrx_C2.1 **R**	TGCCCGACCTCCGTTTTATG
AtNIP1.1 F	GCCAACTCTTGGTGCGATTG
AtNIP1.1 R	TGCTACCGATTCTCACGGTC
AtNIP2.1 F	CAACACTTGGTGCCGTCTCT
AtNIP2.1 R	TACAATGAGAGCAGCGAACAGA
AtNIP3.1 F	CCGTGATCGGGGCATTATCA
AtNIP3.1 R	TTGGGAAGCTTCTTGGCGAT
AtNIP5.1 F	ATCATCGGAAACCAACGCCT
AtNIP5.1 R	TCTGGTTCACGATTGGACCG
AtNIP6.1 F	AAGACTCCCTCCGGTCACTT
AtNIP6.1 R	CCCAACAAACTCTGCTCCCA
AtNIP7.1 F	GGAGGATCGATGAACCCAGC
AtNIP7.1 R	GGGAAACTGGAGAAGGGCAA

Supplementary Table2. ICP-MS operating conditions

RF Power	1550 W
Plasma argon flow	15 L/min
Nebulizer argon flow	1.1 L/min
Auxiliary argon flow	1.2 L/min
Monitored ion	As ^{75}
Dwell Time	500 ms
